# Antigen Transfer from Exosomes to Dendritic Cells as an Explanation for the Immune Enhancement Seen by IgE Immune Complexes

**DOI:** 10.1371/journal.pone.0110609

**Published:** 2014-10-20

**Authors:** Rebecca K. Martin, Keith B. Brooks, Frida Henningsson, Birgitta Heyman, Daniel H. Conrad

**Affiliations:** 1 Department of Microbiology and Immunology, Virginia Commonwealth University, Richmond, Virginia, United States of America; 2 Department of Medical Biochemistry and Microbiology, Biomedical Center, Uppsala University, Uppsala, Sweden; King's College London, United Kingdom

## Abstract

IgE antigen complexes induce increased specific T cell proliferation and increased specific IgG production. Immediately after immunization, CD23^+^ B cells capture IgE antigen complexes, transport them to the spleen where, via unknown mechanisms, dendritic cells capture the antigen and present it to T cells. CD23, the low affinity IgE receptor, binds IgE antigen complexes and internalizes them. In this study, we show that these complexes are processed onto B-cell derived exosomes (bexosomes) in a CD23 dependent manner. The bexosomes carry CD23, IgE and MHC II and stimulate antigen specific T-cell proliferation *in vitro*. When IgE antigen complex stimulated bexosomes are incubated with dendritic cells, dendritic cells induce specific T-cell proliferation *in vivo*, similar to IgE antigen complexes. This suggests that bexosomes can provide the essential transfer mechanism for IgE antigen complexes from B cells to dendritic cells.

## Introduction

Antibody, in complex with its antigen, provides the immune system with a feedback response against the complexed antigen. This feedback mechanism results in immune stimulation or suppression depending upon the antibody class (reviewed in [1]). IgE, complexed with antigen, has long been shown to induce a significantly increased immune response over antigen alone. This response results in increased antigen specific T cell proliferation and antigen specific IgG *in vivo* [2]. The low affinity receptor for IgE (CD23) is traditionally known for its role in the negative regulation of IgE synthesis. But, CD23 has additionally been known to internalize IgE antigen complexes and promote antigen presentation. Extensive studies using CD23^−/−^ animals proved CD23 to be essential to the immunostimulatory properties of IgE antigen complexes [2]. Combining antigen presentation properties of CD23 with the observed immunostimulation led to the hypothesis that antigen presentation by CD23^+^ B cells was responsible for these activities. This hypothesis was challenged by the recent finding that B cells rapidly transfer the antigen to the spleen [3] and were dispensable after 4 hours post IgE antigen complex injection. After that period, dendritic cells (DCs) were required [4]. These intriguing findings suggested that IgE antigen complexes or fragments thereof were being carried to the secondary lymphoid system, in this case the spleen, by CD23^+^ B cells and then were being transferred to DCs. This led to the obvious question, what is the mechanism of this transfer? *Henningson et al* [4] suggested two possibilities; trogocytosis by DCs or exosomal mediated transfer from B cells to DCs.

Exosomes are defined as tiny membrane bound particles ranging from 30–150 nm. Although originally discovered in the 1980 s and thought to be primarily for cellular waste [5], exosomal research has undergone a resurgence given the protein and micro-RNA cargo that is packed into these particles (reviewed in [6]). In a previous publication, we demonstrated that CD23 and its protease, ADAM10 were found in B cell derived exosomes (bexosomes) from both mouse and human B cells [7]. Additionally, β_2_-adrenergic stimulation of B cells further increased both CD23 and ADAM10 levels in bexosomes [8]. In this study we show that B cells stimulated with anti-CD40 and IL-4, in the presence of IgE antigen complexes, release bexosomes that contain both CD23 and IgE. In agreement with earlier studies [9], these bexosomes were capable of directly stimulating antigen specific T cells *in vitro*, presumably via the MHC peptide complexes found on these bexosomes. Intriguingly, this stimulation was greatly enhanced if the bexosomes were isolated from B cells stimulated in the presence of IgE antigen complexes, and this enhanced stimulation was CD23 dependent. This data supports earlier *in vitro* studies where B cells were shown to directly present IgE antigen complexes, taken up via CD23, to T cells [10]. Yet, *in vivo*, direct injection of bexosomes did not cause increased T cell proliferation, which agrees with the requirement of DCs [4]. In contrast, culture of bexosomes with bone marrow derived DCs (BMDC), followed by isolation and injection of DCs did directly enhance *in vivo* antigen specific T cell proliferation. Overall, this supports the hypothesis that bexosomes are responsible for antigen transfer from B cells to DCs, thus, providing a mechanism and suggesting a model to explain the importance of DCs in the immunostimulatory activity of IgE complexes.

## Materials and Methods

### Mice

Mice were maintained in the Virginia Commonwealth University animal facility in accordance to guidelines for the humane treatment of laboratory animals set forth by the National Institutes of Health and the American Association for the Accreditation of Laboratory Animal Care. C57BL/6 and BALB/C mice were purchased from Jackson Labs; DO11.10 and OTII mice were progeny from breeding pairs purchased from Jackson Laboratory. CD23 knockout (BALB/C) [11] or B cell specific ADAM10 deficient mice (ADAM10^B−/−^)(BALB/C backcross at least 6 generations) [12] were bred in house. Mice were euthanized with isoflurane inhalation, followed by cervical dislocation. All mouse protocols were approved by the Virginia Commonwealth University Institutional Animal Care and Use Committee, approval numbers are AM10065 and AM10269.

### Exosomal Cell Cultures

All fetal bovine serum (FBS) used in exosome cultures was first centrifuged at 100,000xg for 2 hours to deplete bovine exosomes. Splenic B cells were isolated using B220 positive magnetic bead selection as per manufacturer protocol (Miltenyi Macs system) and cultured for 3 days in 6 well plates at 1×10^6^ cells/mL in cRPMI 1640 [13] containing 1 µg/mL anti-CD40 (HM40-3) (Biolegend) and 10,000 U/mL IL-4 (gift from Bill Paul NIH). When indicated, 10 µg/ml of anti-TNP- IgE (IGELb4) [14] was present. When larger amounts of exosomes were needed, B lymphoma line M12.4.5 (M12) [15] was used. M12 cells (1×10^6^cells/mL – total cell number 3–5×10^8^) were cultured for 24 hrs in cRPMI 1640/10% FBS (exosome-free as described previously) containing anti-CD40, IL-4, and anti-TNP (IGELb4) or anti-DNP IgE (10 µg/mL) [14], [16].

### Exosomal Isolation

Exosomes were isolated as previously described [17]. Briefly, apoptotic bodies in cell free supernatants were removed by centrifugation at 27,000×g for 20 minutes. Finally, exosomes were harvested by spinning at 100,000×g for 1 hour; the exosome pellet was resuspended in 5 mL of HBS plus 2mM Ca^2+^ and pelleted again at 100,000×g for 1 hour. Final exosome pellet were resuspended in HBS plus 2mM Ca^2+^ and then passed through a 0.2 µm filter to remove particles larger than 200 nm and to assure sterility. Bradford Assay exosome yields were approximately 1.0–1.62 µg/µL, from B cell cultures and about 10-fold higher with M12 cultures depending upon original cell number.

### Western Blotting

Western Blots were performed using 10% Bis-Tris gels and a mini gel system (Life Technologies). Equal amounts of exosome protein (20 µg) were loaded. NuPage MES SDS Running Buffer and NuPAGE MES Transfer Buffer were used according to manufacturer’s instructions. After electrophoresis and transfer to nitrocellulose paper, blocking was performed using 5% powdered milk solution/0.05% v/v Tween-20 (two hours) followed by the primary antibody incubation (1 hour minimum) followed by HRP secondary antibody. Antibodies used were as follows: Mouse anti-mouse MHCII H2-I/Adβ (5K43) (Santa Cruz); Rabbit anti-mouse IgE (Fc specific) (Acris); goat anti-rabbit HRP (Southern Biotech); Rabbit anti-mouse CD23 as described [18]; Rabbit anti-CD9 (polyclonal) (Sigma).

### Specific T cell proliferation

#### *In vitro*

DO11.10 T cells were isolated using magnetic bead selection (Miltenyi Macs)B220^+^, CD11b^+^ and CD11c^+^ cells were depleted and then CD4 positive T cells were isolated using L3T4, non-activating selection. Bexosomes (15–20 µg/100 µL) were added to 1×10^5^ DO11.10 T cells in a final volume of 200 µL cRPMI/well in 96 well plates; TNP or DNP-OVA was added in indicated amounts. After 96 hours, cells were pulsed with 1 µCi/well of [H3]-thymidine for 24 hours (Perkin Elmer). Plates were then harvested using a Filtermate cell harvester onto GFC plates. Assays were read using a Topcount Plate Counter (Perkin Elmer, Waltham, MA).

#### *In vivo*

(B cell specific ADAM10 deficient) ADAM10^B−/−^BALB/C or WT BALB/C mice were injected with IgE immune complexes (20 µg TNP-OVA +50 µg anti-TNP-IgE [4]. For bexosome or BMDC derived dexosome experiments, bexosomes were added to the DC cultures for 24 hours. After washing, LPS was added and cultures were continued for 72 hours and dexosomes were then isolated by centrifugation. Dexosomes, BMDCs or bexosomes or were i.v. injected into BALB/C mice that had been adoptively transferred (AT) with DO11.10 total splenocytes 24 hours previous. After 3 days, DO11.10 cell numbers in the spleen were determined either by flow cytometry or immunohistochemistry as described [4].

### Flow Cytometry/Immunohistochemistry

Single cell suspensions were labeled with antibodies for cytometric analysis. Antibodies included unlabeled anti-mouse FcγR (2.4G2), FITC-conjugated anti-mouse D011.10 (KJ1-26) (Biolegend), and PE-conjugated anti-mouse CD4 (145-2C11) (Biolegend). Flow cytometric analysis was performed using the Canto (BD Biosciences). Data analysis was conducted using FlowJo software v7.6.5 (Tree Star). Sections were prepared from frozen tissue and fixed with cold Acetone. Sections were blocked using 5% normal horse serum plus 1.5% BSA. Sections were stained using Biotinylated anti-DO11.10 (KJ1-26) followed by Streptavidin-APC and anti-B220-PE (RA3-6B2) (Biolegend). Slides were cover slipped utilizing Vectashield Hardset (Vector Labs) and imaged on the Leica TCS-SP2 AOBS CLSM Confocal laser-scanning microscope.

### BMDC culture

Mouse bone marrow-derived dendritic cells (BMDC’s) were derived from femurs of WT mice and cultured in complete RPMI (cRPMI) 1640 containing 10% FBS, 2mM L-glutamine, 100 U/mL penicillin, 100 µg/mL streptomycin, 1mM HEPES (Quality Biological, Inc.), and 1mM sodium pyruvate (Cellgro). Cultures were supplemented with IL-4 1 ng/mL (Peprotech) and GM-CSF 50 ng/mL (Peprotech). Mature BMDC were harvested after 6 days of culture [9].

### Statistical Analysis

When dealing with two groups, p-values were calculated using unpaired two-tailed Student’s t-tests in GraphPad Prism. Error bars represent the standard deviation (SD) between samples. p<0.05 is considered significant.

## Results and Discussion

### IgE is associated with bexosomes in a CD23 dependent manner

**Figure 1:**
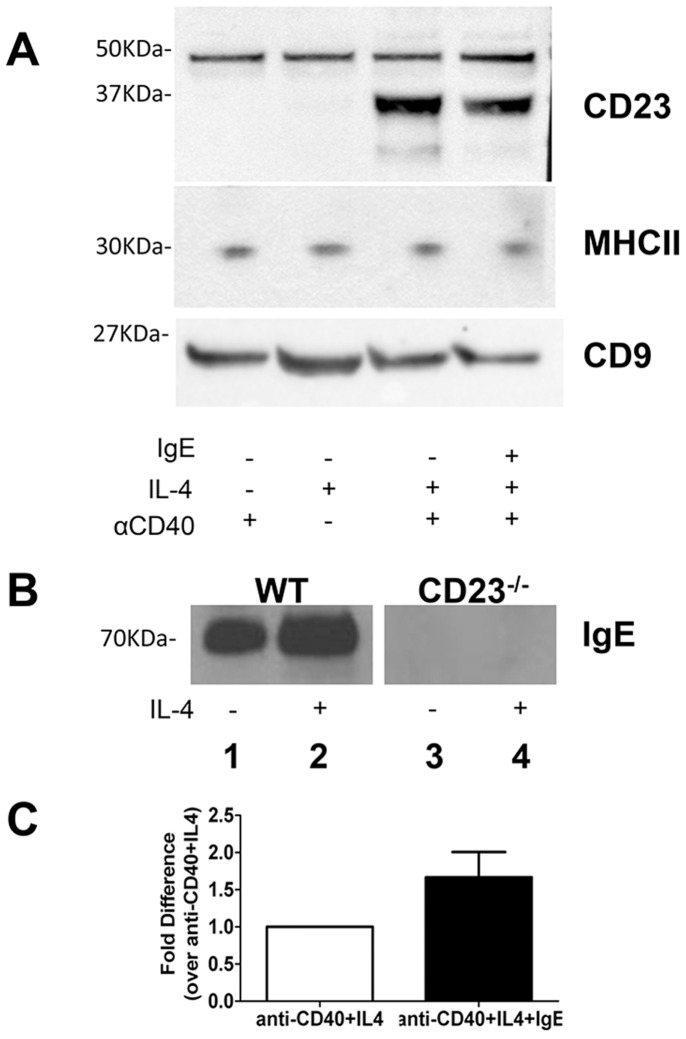
IgE and CD23 are associated with bexosomes. (A) Bexosomes were isolated from B cell cultures stimulated with anti-CD40± IL-4 or IgE as indicated. 1.2511 fold increase ±0.177SD between lanes three and four. Equal bexosome protein was loaded on each lane and the blot was probed with polyclonal anti-CD23. Blot was probed with anti-MHC class II or anti-CD9and the result is shown at the bottom of (A). (B) Exosomes from B cell cultures stimulated with anti-CD40 and IgE (All lanes). IL-4 stimulation is as indicated. Lanes 1 and 2 are bexosomes from WT mice, Lanes, 3 and 4 are bexosomes from CD23^−/−^ mice. Blots are representative of at least three independent experiments. (C) Graph represents fold difference in densitometry between anti-CD23 blots for anti-CD40/IL4 and anti-CD40/IL4/IgE.

As mentioned, CD23 and ADAM10 are both found associated with bexosomes [7]. In order to determine if IgE is additionally associated, B cells were stimulated with anti-CD40± IL-4 in the presence of IgE/Ag ICs. After 48 hours of culture, bexosomes were isolated by centrifugation. Equal amounts of protein were analyzed by Western blotting. Fig. 1A,C shows that IL-4 stimulated B cells increases bexosomal CD23 levels and that IgE addition increases CD23. The blot indicated equal MHC class II expression. Interestingly, MHC class II levels were not increased by IL-4, in contrast to the well-known cell surface increase induced by this cytokine. The bexosomes were additionally blotted for the tetraspanin CD9, which is an exosomal marker (Fig. 1A). In an earlier study using LPS as the activator [7], we found that ADAM10 was needed for bexosomal CD23 expression. With anti-CD40, CD23 is present even if ADAM10^−/−^ B cells are used; although the CD23 expression level is lower (data not shown). This indicates that CD40 activation is more efficient in allowing CD23 incorporation into bexosomes. In Fig. 1B, the blot is examined using an ε-specific anti-IgE antibody, and lane 1 shows a strong 85Kd band indicates the presence of IgE. The amount of IgE was additionally increased in the presence of IL-4 (lane 2). This band was not present when B cells lacking CD23 were used (lanes 3 and 4) regardless of IL-4 stimulation. Thus, CD40-mediated CD23^+^ B cell activation results in release of bexosomes that contains CD23 and bound IgE.

### Increased *in vivo* antigen specific T cell proliferation is seen with bexosomes from anti-CD40/IL4 activated B cells when IgE/antigen complexes are present in the culture

**Figure 2:**
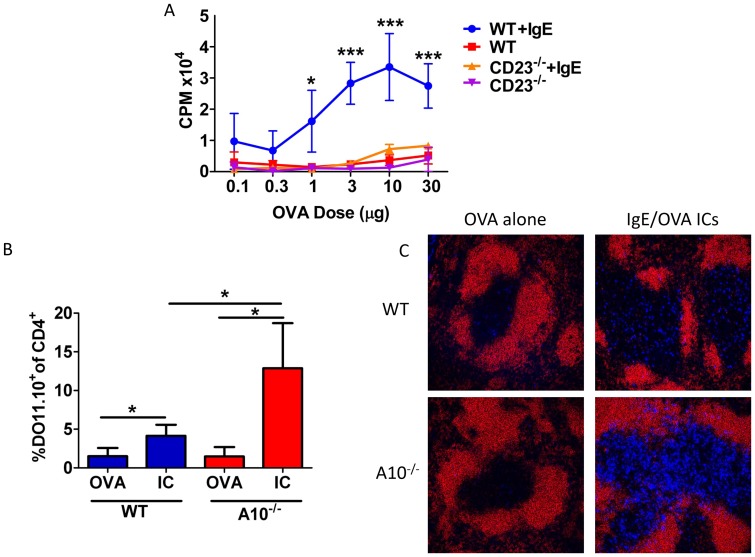
Bexosome-induced antigen specific T cell proliferation is enhanced by IgE, only in the presence of CD23and in vivo IgE immune complex T proliferation is enhanced in ADAM10B^−/−^ mice. (A) B cell cultures were stimulated as in *Methods*. Stimulated B cells from either WT or CD23^−/−^ mice were pre-incubated ±IgE for 24 hours and then IgE/Ag ICs were added for an additional 24 hours. Bexosomes were isolated and cultured with purified Ag-specific DO11 T cells for 3 days. Proliferation was determined using a [3H]-thymidine pulse (*Methods*). (B,C) On day −1 WT or ADAM10^B−/−^ mice were adoptively transferred with Ag-specific DO11 T cells and on day 0 immunized with IgE/Ag ICs (*Methods*); on day 3, spleens were removed and examined for expanded Ag specific DO11^+^ CD4^+^ T cells by flow cytometry (B) or immunohistochemistry (C). *p>0.05, ***p>0.0005; n = at least five per group.

As mentioned above, bexosomes are known to induce antigen specific T cell proliferation if isolated from B cells cultured in the presence of the specific antigen [9]. The finding (Fig. 1) that CD23 and IgE are present in bexosomes suggests that MHC class II peptide loading should be enhanced when IgE complexes are included in the culture. That this is indeed true is shown in Fig. 2A where the initial B cells were cultured with increasing amounts of antigen (TNP-OVA) alone or the same plus anti -TNP-IgE. With B cells from either BALB/C or C57BL/6 mice and the corresponding strains of OVA-specific T cells, DO11.10 (Fig. 2A) or OTII (Fig. S1), increased specific T cell proliferation is seen when antigen specific IgE is present in the cultures. This data looks quite similar to the studies that initially demonstrated the enhanced antigen-presentation by IgE complexes with CD23^+^ B cells [10] and relates to enhanced MHC class II loading due to the increased antigen found in the endosomal compartments. Confirmation that both IgE and CD23 are necessary for the bexosomal induced T cell proliferation is shown in Fig. 2A where increased T proliferation is only seen when both are present. To determine whether ADAM10 was also needed for the enhancement of presentation, we turned to *in vivo* studies. WT or B cell-specific ADAM10 deficient (ADAM10^B−/−^) BALB/C mice were *i.v.* injected on day −1 with 30 million DO11.10 total splenocytes. On day 0, they were *i.v*. injected with anti-TNP IgE/TNP-OVA complexes (Methods). Three days later, OVA-specific T-cell proliferation was determined by flow cytometry and immunohistochemistry. WT mice injected with IgE/Antigen complexes had increased DO11.10 proliferation, as indicated by the number of antigen specific T cells in the spleen, as compared to WT OVA injected (Fig. 2B). This was as expected. But, the ADAM10^B−/−^ mice had significantly more DO11.10 proliferation than did the WT. This finding was further confirmed by immunohistochemistry, where proliferation differences generally look more pronounced. The enhanced OVA-specific DO11.10 cell number in the T cell region of the spleen of the ADAM10B^−/−^ was clearly evident (Fig. 2C). Thus, B cell ADAM10 is clearly not necessary for IgE antigen complex stimulation since this enhancement is seen. As the ADAM10^B−/−^ mice have been shown to have enhanced CD23 expression [19], we hypothesize that the increased CD23 leads to still further IgE-antigen incorporation with enhanced MHC class II loading in the endosomal compartment, leading to the results shown in Fig. 2B and C. These studies also indicate that marginal zone B cells are not necessary for IgE-antigen complex immunostimulation, since marginal zone B cells are absent in ADAM10^B−/−^ mice [19]. It also agrees with the observation that IgE complexes were found bound to mainly follicular B cells and not to MZ B cells [4].

### Bexosomes must first go through DCs in order to stimulate T cells *in vivo*

**Figure 3:**
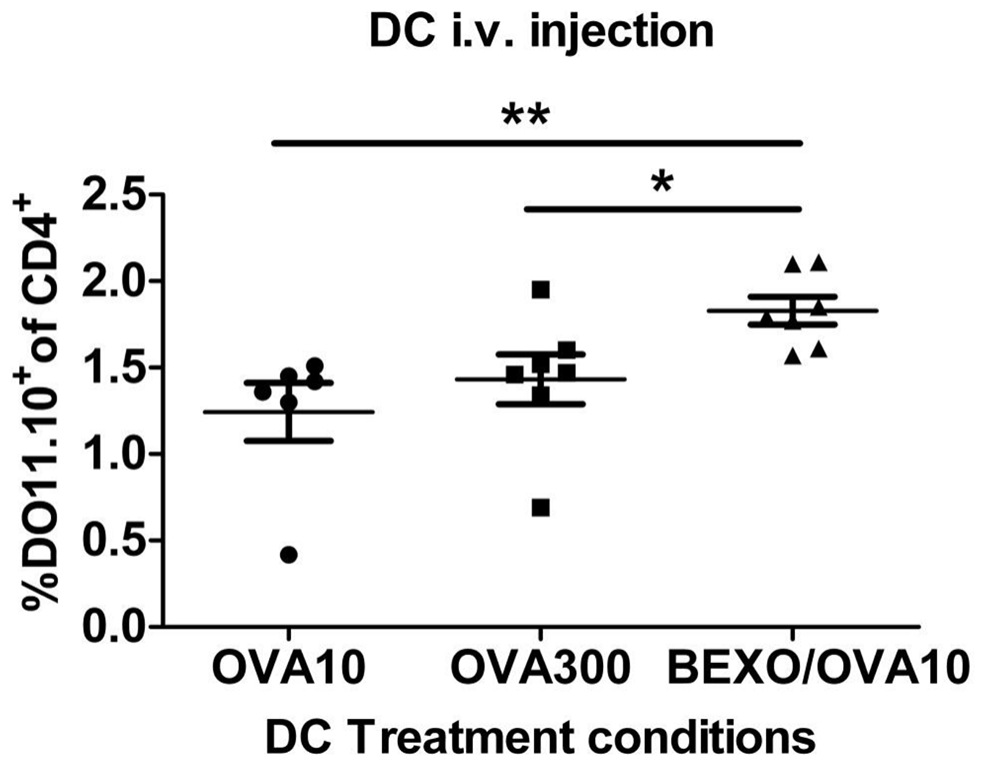
Increased *in vivo* T proliferation with *i.v*. injected DCs incubated with bexosomes. *In vitro* differentiated LPS activated DCs were incubated with bexosomes stimulated with IgE/Ag ICs plus 10 µgOVA, 10 µgOVA or 300 µg OVA for three days. DCs were harvested and equivalent cells were injected into mice AT with DO11 cells. Spleens were assessed by flow cytometry on Day 3. Percent DO11TgTCR+ cells out of total CD4+ T cells were plotted. *p>0.05; n = 5 per group.

**Figure 4:**
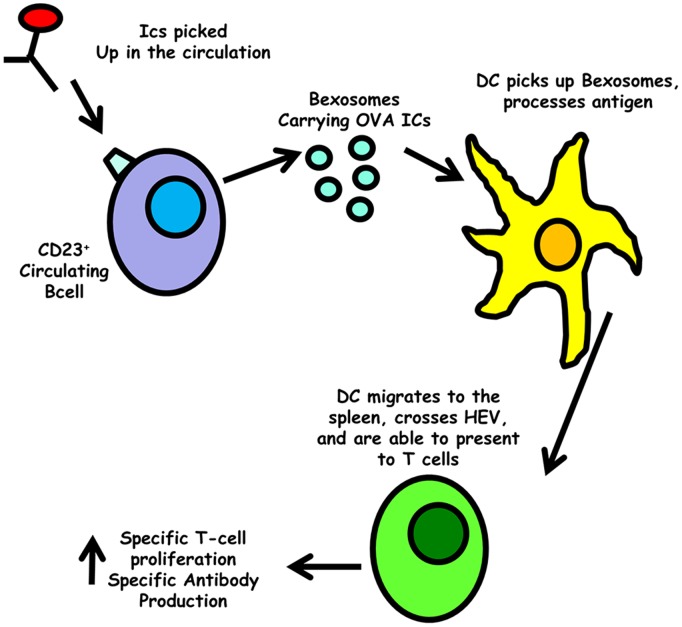
Model for the mechanism of IgE immune complex mediated humoral immunity enhancement. IgE immune complexes are picked up by CD23^+^ B cells and this allows efficient transfer into the spleen. Once there, bexosomes are released and picked up by DCs which then activate antigen specific T cells and subsequently give the enhanced humoral response that is seen in IgE antigen complex injected animals.

To directly test if bexosomes from B cells activated with anti-CD40 and IL-4 would stimulate T cells *in vivo,* bexosomes were isolated from B cells activated as in Fig. 2A with IgE/OVA complexes. They were injected into BALB/C mice AT with OVA-specific DO11.10 cells. This was ineffectual, so to increase the level of bexosomes for injection, 3×10^8^ M12.4.5 cells were activated instead of primary B cells. Bexosomes were isolated and injected into the OVA-specific T cell AT mice. In neither situation did we observe increased levels of OVA-specific T cell proliferation when the spleen was examined 3 days post bexosome injection (data not shown). We considered that the most likely reason for this failure was that the bexosomes were not efficiently crossing the HEV to enter the peri-arteriolar lymphoid sheath (PALs) tissue. To increase chance for HEV crossing, bexosomes were cultured with LPS activated BMDCs, and DC were isolated 3 days post culture with bexosomes. In contrast to directly injected bexosomes, passage of the bexosomes through DCs, with injection of the DCs did cause a small but significant increase in OVA-specific proliferation, as determined by flow cytometry (Fig. 3). This increase was significantly higher than what was seen by control DCs isolated from LPS activated cultured with the same amount of TNP-OVA alone or increased amounts. While the increase in T cell proliferation was not as dramatic as seen when directly injecting IgE immune complexes (Fig. 2B,C), the results are clearly compatible with exosomes being responsible for the transfer of antigen to DCs as described by *Henningsson et al* [4]. Thus, we propose the following model (Fig. 4): IgE immune complexes are picked up by CD23^+^ B cells and this allows efficient transfer into the spleen. Once there, bexosomes are released and picked up by DCs which then activate antigen specific T cells and subsequently give the enhanced humoral response that is seen in IgE antigen complex injected animals [10]. Future studies could examine the mechanism of CD23^+^ bexosomal release and ways to increase transfer of the bexosome incorporated MHC class II cargo to DCs. If successful, this would allow immunization strategies that take advantage of the IgE immune complex stimulation that would bypass any difficulties seen by FcεRI interaction with IgE and antigen, and still allow efficient sensitization with low amounts of antigen.

## Supporting Information

Figure S1 **Bexosome-induced antigen specific T cell proliferation is enhanced by IgE in C57BL/6 model of OVA-specific T cell proliferation.** B cell cultures stimulated as in *Methods*. B cells were incubated with IgE for 24 hours and then IgE/Ag ICs were added. Bexosomes were isolated. B cells were from WT C57BL/6 mice. Bexosomes and purified Ag-specific OTII T cells were cultured for 3 days and proliferation determined using a [3H]-thymidine pulse (*Methods*).
(DOCX)
