# Supplementary material for: Antigen Transfer from Exosomes to Dendritic Cells as an Explanation for the Immune Enhancement Seen by IgE Immune Complexes
Source: PLoS One. 2014 Oct 20;9(10):e110609. doi: 10.1371/journal.pone.0110609 (PMC4203810; doi:10.1371/journal.pone.0110609)
Supplement: Figure S1 — Bexosome-induced antigen specific T cell proliferation is enhanced by IgE in C57BL/6 model of OVA-specific T cell proliferation. B cell cultures stimulated as in Methods. B cells were incubated with IgE for 24 hours and then IgE/Ag ICs were added. Bexosomes were isolated. B cells were from WT C57BL/6 mice. Bexosomes and purified Ag-specific OTII T cells were cultured for 3 days and proliferation determined using a [3H]-thymidine pulse (Methods). (DOCX) [file pone.0110609.s001.docx]

#####

#####
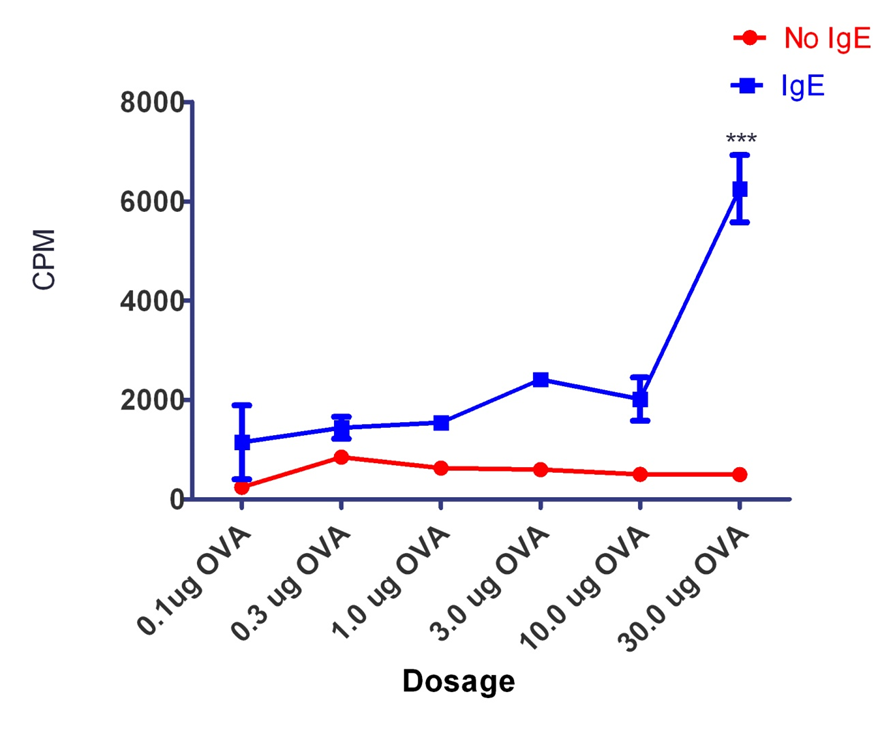


##### Supplemental Figure 1. Bexosome-induced antigen specific T cell proliferation is enhanced by IgE in C57BL/6 model of OVA-specific T cell proliferation

B cell cultures stimulated as in *Methods*. B cells were incubated with IgE for 24 hours and then IgE/Ag ICs were added. Bexosomes were isolated. B cells were from WT C57BL/6 mice. Bexosomes and purified Ag-specific OTII T cells were cultured for 3 days and proliferation determined using a [3H]-thymidine pulse (*Methods*).
